# Supplementary material for: Seminoma and Embryonal Carcinoma Footprints Identified by Analysis of Integrated Genome-Wide Epigenetic and Expression Profiles of Germ Cell Cancer Cell Lines
Source: PLoS One. 2014 Jun 2;9(6):e98330. doi: 10.1371/journal.pone.0098330 (PMC4041891; doi:10.1371/journal.pone.0098330)
Supplement: File S1 — ZIP file containing DMRforPairs output for significant regions. Please start from the html files. (ZIP) [file pone.0098330.s008.zip › figures/12061.pdf]

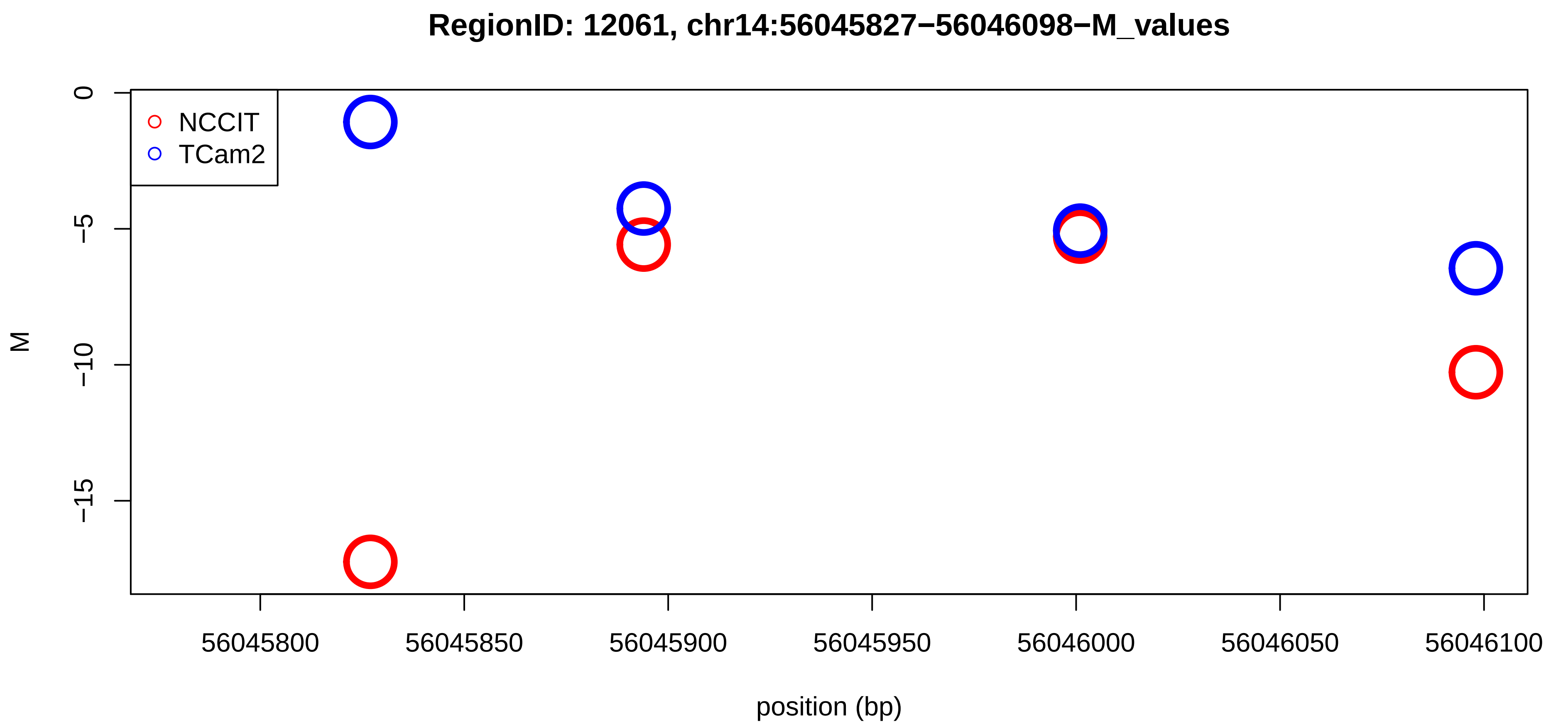
RegionID: 12061, chr14:56045827−56046098−M_values
M
0
−5
−10
−15
NCCIT
TCam2
56045800
56045850
56045900
56045950
56046000
56046050
56046100
position (bp)

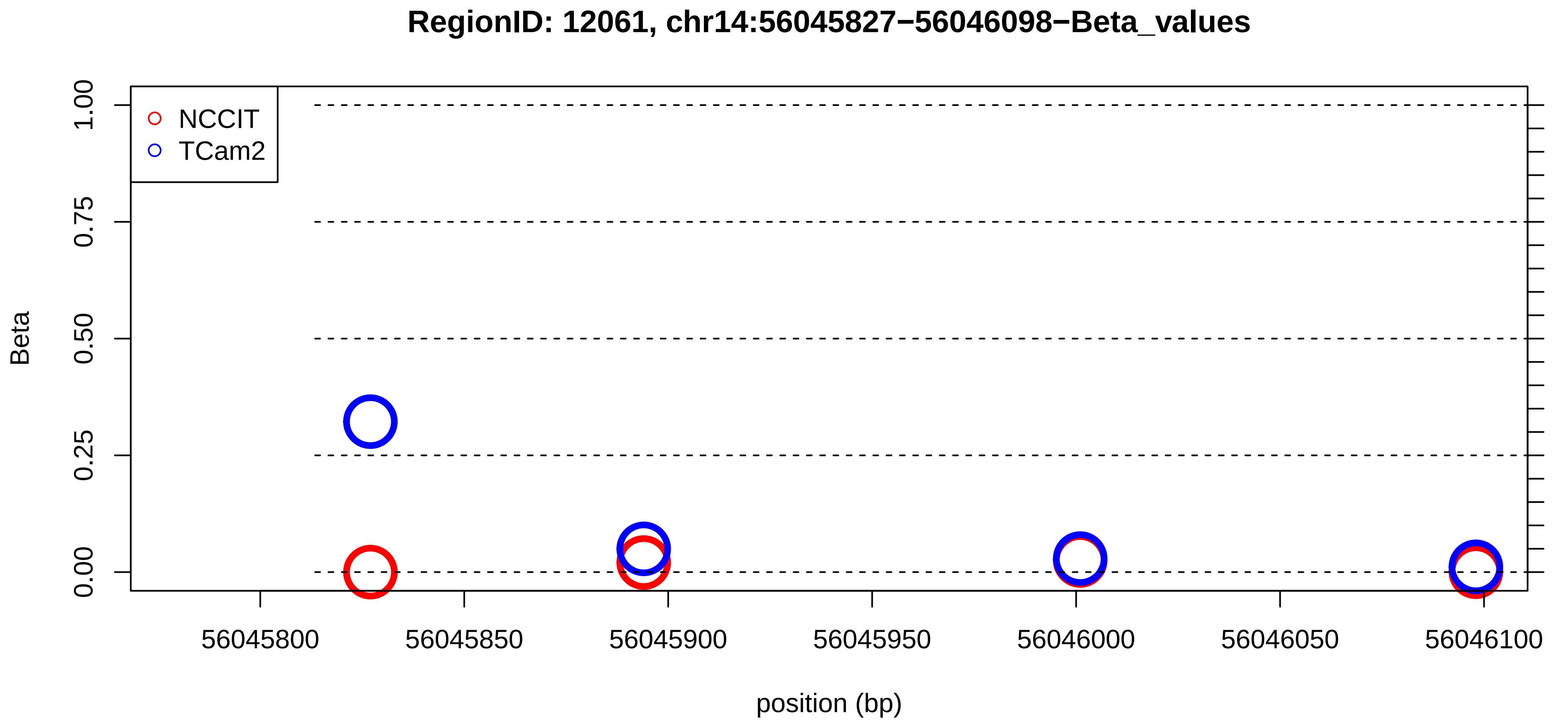
RegionID: 12061, chr14:56045827−56046098−Beta_values
Beta
1.00
0.75
0.50
0.25
0.00
NCCIT
TCam2
56045800
56045850
56045900
56045950
56046000
56046050
56046100
position (bp)
